# Formulation and Systematic Optimisation of Polymeric Blend Nanoparticles via Box–Behnken Design

**DOI:** 10.3390/pharmaceutics17101351

**Published:** 2025-10-20

**Authors:** Basant Salah Mahmoud, Christopher McConville

**Affiliations:** 1 School of Pharmacy, College of Medical and Dental Sciences, University of Birmingham, Birmingham B15 2TT, UK; bsm465@alumni.bham.ac.uk; 2 Hormones Department, Institute of Medical Research and Clinical Studies, National Research Centre, El Buhouth St., Dokki, Cairo 12622, Egypt; 3 Centre for Genomic Medicine, School of Biomedical Sciences, Ulster University, Coleraine BT52 1SA, UK

**Keywords:** polycaprolactone, polylactic-co-glycolic acid, irinotecan hydrochloride, nanoparticles, Box–Behnken design, size, zeta potential, encapsulation efficiency

## Abstract

**Background/Objectives:** Despite the advantages of polycaprolactone (PCL) for drug delivery, it still lacks effective approaches to enhance its encapsulation of drugs. Blending PCL with less hydrophobic polymers can tailor physicochemical properties to overcome these limitations. This study, for the first time, integrates two beneficial approaches—polymer blending and Box–Behnken design (BBD) optimisation—to develop PCL-based blend nanoparticles (NPs) with enhanced encapsulation efficiency (EE), controlled particle size, and improved stability through surface charge modulation. **Methods:** Drug-loaded blend NPs were developed using a double emulsion method, with different polymer ratios. A BBD model was employed to investigate the influential factors that control the size, charge, and EE. **Results:** Blending PCL with a less hydrophobic polymer significantly improved EE, achieving 60.96% under optimal conditions. The BBD model successfully predicted conditions for obtaining NPs with optimum size, negative charge, and enhanced drug encapsulation. The drug amount was identified as the most influential factor for EE, while polymer amounts significantly impacted size and charge. **Conclusions:** Careful control of polymer ratios, drug amount, and surfactant levels was shown to significantly influence particle size, surface charge, and EE, with the balanced 50:50 PCL:PLGA blend achieving optimal physicochemical performance. Using the BBD, the study identified the predicted optimal formulation consisting of 162 mg polymer blend, 8.37 mg drug, and 8% surfactant, which is expected to yield NPs with a size of 283.06 nm, zeta potential of −31.54 mV, and EE of 70%. The application of BBD allowed systematic evaluation of the factors and their interactions, providing robust predictive models.

## 1. Introduction

Nanomedicine has the potential to make revolutionary leaps over traditional chemotherapy in cancer treatment, especially brain cancer, which possesses extra complexity added by the blood–brain barrier (BBB) [1]. Nanoparticles (NPs) can be designed with specific characteristics for their intended purpose, whether as diagnostic tools or drug carriers, protecting the normal cells from toxic drugs and protecting drugs from early metabolism and off-site accumulation [2]. Ligands specific to cell surface receptors can be attached to the surface of the NPs to enable directed localisation of the NPs by active targeting or by passive targeting by exploiting the enhanced permeability and retention effect [3]. Polymeric NPs possess several advantages by being safe and biologically tolerable due to their low reactivity and biodegradability [4]. They also possess the advantage of controlled release and the capacity to protect drugs from the surrounding media. This allows them to be designed as drug-loaded vehicles easily manipulated and tailored to fit the intended purpose.

Polycaprolactone (PCL) is a semi-crystalline hydrophobic polymer. It is an inert, non-toxic polymer that is advantageous as a carrier for a wide array of drugs, allowing drug delivery and sustained drug release due to its slow biodegradation. However, its crystallinity limits its ability to encapsulate high drug concentrations, specifically hydrophilic drugs that tend to avoid hydrophobic cores and favour amorphous regions during the process of development [5]. Previous studies have predominantly focused on single-polymer systems to produce drug-loaded NPs, but such approaches often result in suboptimal encapsulation and lack systematic optimisation. Research exploring strategies to improve the encapsulation efficiency (EE) of semicrystalline polymers as PCL remains scarce. To address this gap, the present work introduces, for the first time, a Box–Behnken design (BBD) optimisation of polymer-blend nanoparticles aimed at enhancing the EE of PCL while enabling precise control over particle size and surface charge.

One of the approaches to alleviate the issue of PCL’s poor encapsulation of hydrophilic drugs is by forming blends with less hydrophobic polymers. Polymer blends are favourable due to their mixed properties, which are not usually present in individual polymers [6]. Blended polymers can have their properties tailored to overcome the limitations of the individual polymers used for drug delivery [7]. The physicochemical characteristics of polymers, such as their chemical and crystal structures, solubility, degradation, and hydrophobicity, impact the surface charge, drug compatibility, and release patterns in pharmaceutical formulations. Such factors also influence the selection of a suitable polymer(s) for the intended application [8].

In this context, blending a semi-crystalline polymer such as PCL with an amorphous polymer has emerged as an effective approach to improve drug encapsulation. The selection of an amorphous polymer such as polylactic-co-glycolic acid (PLGA) can be influenced by the ratio of its glycolic and lactic acid monomers, which impacts its solubility and degradation [9]. PLGA 50/50 was reported to have a faster degradation and erosion rate compared to PLGA 75/25 because of a greater rate of hydrolysis for the lactide/glycolide monomers. PLGA chains also hydrolyse faster than PCL chains in PLGA/PCL/PLGA triblock copolymers, as previously reported [10]. Therefore, PLGA 50/50 was selected in this study to form blends with different ratios of PCL.

Irinotecan hydrochloride (IRH) was selected as the model drug for this study because it represents a challenging yet clinically relevant hydrophilic chemotherapeutic agent. IRH acts as a topoisomerase I inhibitor, preventing DNA replication and promoting apoptosis, with its mechanism of action being independent of MGMT methylation status—making it effective against various resistant tumour types [11]. Moreover, both IRH and its highly active metabolite, SN-38, are not substrates of the P-glycoprotein efflux transporter, allowing them to bypass multidrug resistance [12]. Despite these therapeutic advantages, IRH faces significant formulation challenges: it exhibits limited permeability across the BBB due to its relatively high molecular weight (677 Da) and hydrophilicity, and it is chemically unstable at physiological pH, where the active lactone form readily hydrolyses into the inactive carboxylate form [13,14]. These stability and permeability limitations significantly reduce its therapeutic potential. Therefore, developing an optimised NP system to encapsulate and protect IRH provides a valuable model for improving the stability and bioavailability of hydrophilic drugs with similar physicochemical characteristics.

Optimisation of the formulation parameters of the blended polymers is required to obtain an effective formulation with suitable size, charge, and EE for drug delivery applications [7]. A BBD of Experiments is a strategic approach for optimising complex formulations and understanding the influence of multiple variables on their critical quality attributes. Therefore, a BBD was performed in this paper to examine and better understand the effects of the independent variables on key characteristics of PCL/PLGA blend NPs.

## 2. Materials and Methods

### 2.1. Chemicals

The DL-lactide/Glycolide copolymers; PURASORB^®^ PDLG 5002A (50% Lactide, inherent viscosity of 0.2 dl/g) were purchased from Corbion Purac (Gorinchem, The Netherlands). PCL (14,000–50,000 g/mol), polyvinyl alcohol (PVA) (13,000–23,000 g/mol), sucrose, sodium chloride (NaCl), potassium dihydrogen phosphate, octane-1-sulfonic acid, and orthophosphoric acid were purchased from Sigma-Aldrich (Gillingham, Dorset, UK). IRH was purchased from LGM Pharma (Erlanger, KY, USA). Acetonitrile and dichloromethane (DCM) were purchased from ThermoFisher Scientific (Loughborough, UK).

### 2.2. Development of PCL-PLGA Blend NPs by Double Emulsion (Water in Oil in Water (W/O/W)) Solvent Evaporation Technique

Drug-loaded PCL-PLGA blends were developed using the double emulsion method, with different amounts of PCL to PLGA at 25:75, 50:50, and 75:25 ratios. Initially, PCL was added to DCM and stirred at 600 rpm until completely dissolved. Then, the required amount of PLGA was added and stirred until fully solubilised to form the oil phase (O). The required amount of IRH was dissolved in 3 mL dH_2_O containing 10 mg/mL NaCl to prepare the internal aqueous phase (W1). Using a burette, W1 was added dropwise (60 drops/min) to the organic phase, under gentle stirring, to create the primary emulsion. This emulsion was then poured into the external aqueous phase (W2), which consisted of 25 mL of PVA (%*w/v*) surfactant solution containing 2.5% NaCl, to form the double emulsion. The resulting mixture was sonicated in an ice bath (to prevent overheating by the probe sonicator) for 10 min, followed by solvent evaporation under gentle stirring at room temperature. The NPs were then collected by centrifugation at 24,500 rpm using Beckman Coulter centrifuge (Beckman Coulter Ltd, High Wycombe, Buckinghamshire, UK) for 30 min. The conditions have been maintained at room temperature throughout the development process. The free drug was removed by washing the NPs twice with dH_2_O. Then, freezing occurred overnight at −80 °C in 5% sucrose solution. Lyophilisation then took place at 0.01 mbar and −85 °C for 48 h using Labconco lyophiliser (Labconco Corporation, Kansas City, MO, USA). The lyophilised NP formulations were then stored in airtight containers at 4 °C until further characterisation.

### 2.3. BBD

A three-factor, three-level BBD was employed using Minitab software version 19.2020.2.0 (Minitab LLC, State College, PA, USA). The design included a total of 15 experimental runs of IRH-loaded PCL-PLGA blend NP formulations, with three replicates at the centre point to enable estimation of experimental error and to assess model reproducibility. All experimental runs were conducted in a randomised order generated by the software to minimise potential bias from uncontrolled variables. The coded and actual levels of the variables are presented in Table 1. Table 2 provides the actual levels corresponding to each of the formulations in BBD. The levels of the independent variables were determined based on preliminary optimisation trials and literature data. The polymer amount (X1, 108 mg) was selected as the midpoint providing sufficient matrix density for encapsulation without increasing viscosity. The drug amount (X2, 6 mg) represented the optimal balance between solubility and EE, as higher concentrations caused precipitation and lower amounts reduced loading. The surfactant concentration (X3, 4% PVA) was chosen to ensure adequate interfacial stabilisation while avoiding excessive viscosity that could hinder emulsification and increase particle size above acceptable levels, while reducing stability.

### 2.4. Measurement of Particle Size and Zeta Potential

Lyophilised NPs (2 mg, *n* = 3) were dispersed in 3 mL dH_2_O and analysed for hydrodynamic diameter, polydispersity index (PDI), and zeta potential using a Zetasizer (Malvern Panalytical Ltd., Malvern, Worcestershire, UK). Size and PDI were measured at 25 °C in disposable polystyrene cuvettes (DTS0012, Malvern Panalytical Ltd, Malvern, Worcestershire, UK), while zeta potential was determined in folded capillary cells (DTS1070, Malvern Panalytical Ltd, Malvern, Worcestershire, UK) under the same conditions. Samples (1 mL) were equilibrated for 120 s before measurement.

### 2.5. Detection of IRH by Ion Pair HPLC Method and Measurement of EE

The analysis of free IRH was conducted by using an Agilent Technologies 1260 infinity II HPLC system (Agilent Technologies, Santa Clara, CA, USA) with a quaternary gradient pump. A C18 column (150 mm × 4.6 cm) with 5 μm particle size was used to perform the separation at 25 °C (ThermoFisher Scientific, Loughborough, UK).

An injection volume of 20 μL, a run time of 10 min, and a flow rate of 1 mL/min were selected for the analysis. The mobile phase was composed of an ion pair solution of 1.2 g octane-l-sulfonic acid in 500 mL dH_2_O (solution A), and 13.6 g potassium dihydrogen phosphate dissolved in 500 mL dH_2_O (solution B). In addition to solutions A and B, acetonitrile was added to obtain a ratio of 30:30:40 *v/v/v* at pH 3, using orthophosphoric acid. The analysis was performed with a UV detector at 265 nm wavelength.

IRH standards were prepared at 4, 10, 30, 40, 50, 80 and 100 µg/mL. Each standard was measured in triplicate. Linearity was assessed by linear regression, and the calibration curve was accepted with R^2^ > 0.99.

The free drug was determined in the supernatant of each NP formulation via HPLC analysis. EE was calculated using the following equation:

(1) $$\mathrm{EE}\;(\%)\;=\frac{Initial\;drug\;amount-Drug\;in\;the\;supernatant}{Initial\;drug\;amount}\times 100$$

### 2.6. Statistical Analysis

Data produced from BBD were fitted using the Minitab software to obtain the best-fit model represented by the R^2^. Analysis of variance (ANOVA) was performed to ensure the model was a good fit. F statistics (*F*-tests) and Probability values (*p*-values) were employed to determine the significance of the regression coefficient. Values < 0.05 were considered significant. Model validation was further performed by comparing predicted and experimental responses, with residuals and percentage error presented. Data fitted to the software were the mean of three replicated measurements. The residuals were small, randomly distributed, and normally distributed, confirming adequacy of the fitted model and supporting its robustness for predicting NP size, charge, and EE. A Pareto chart was used to determine the significant variables. Finally, 2D and 3D plots were used to provide a graphical representation of the model.

## 3. Results

### 3.1. Size, Charge and EE% of Drug-Loaded PCL-PLGA NPs

The highest EE (60.96 ± 6.62%) was achieved with blend F2 (50:50 PCL: PLGA) (Table 3). Therefore, it was selected as a rational formulation midpoint for subsequent optimisation by BBD, not only due to its relatively high EE but also to explore a balanced integration of polymers with distinct crystallinity and hydrophobicity. This balanced composition is expected to enhance processing stability and yield an intermediate degradation behaviour favourable for controlled release, which will be investigated in future studies.

All formulations were characterised for particle size, PDI, zeta potential, and EE%, and the results are summarised in Table 4. The NPs exhibited sizes ranging from 206.03 to 461.33 nm, with PDI values between 0.21 and 0.60, indicating that the formulations were generally monodisperse. The zeta potential values varied from −21.93 to −36.70 mV, suggesting good colloidal stability across formulations. EE ranged from 49.76 to 66.25%, reflecting the influence of polymer, drug, and surfactant concentrations on drug incorporation within the NP matrix. These results provided the experimental dataset used to fit the BBD models and to evaluate the effect of independent formulation variables on the key physicochemical responses.

### 3.2. BBD Predicted Model and Best Fit Regression Coefficient for Size

The particle size responses were fitted via multiple regression to yield a significant second-order polynomial model (Equation (2)), with R^2^ = 0.92, adjusted R^2^ = 0.77, and a non-significant lack of fit (*p* = 0.22). Regression analysis and ANOVA for NP size are summarized in Table 5 and Table 6, respectively.

Size = 363.2 + 42.0 A + 26.9 B − 38.7 C − 60.0 A*A + 34.4 B*B − 37.8 C*C + 14.3 A*B − 29.5 A*C − 13.0 B*C(2)

The model indicates that polymer amount had a significant positive effect on particle size, while surfactant concentration and the polymer-squared term exerted significant negative effects. The highest significant effect was shown to be the polymer amount, with a contribution of 23.30%.

**Figure 1:**
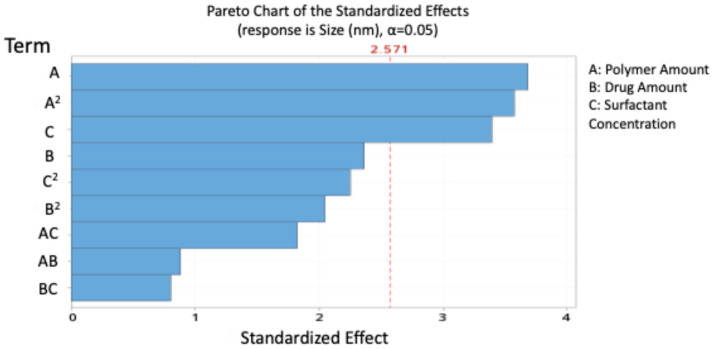
Pareto chart expressing the standardised effect of independent variables and their interaction on the size of the nanoparticles (NPs).

Figure 1 presents the Pareto chart ranking the significant effects of the independent variables on the size of blend NPs.

**Figure 2:**
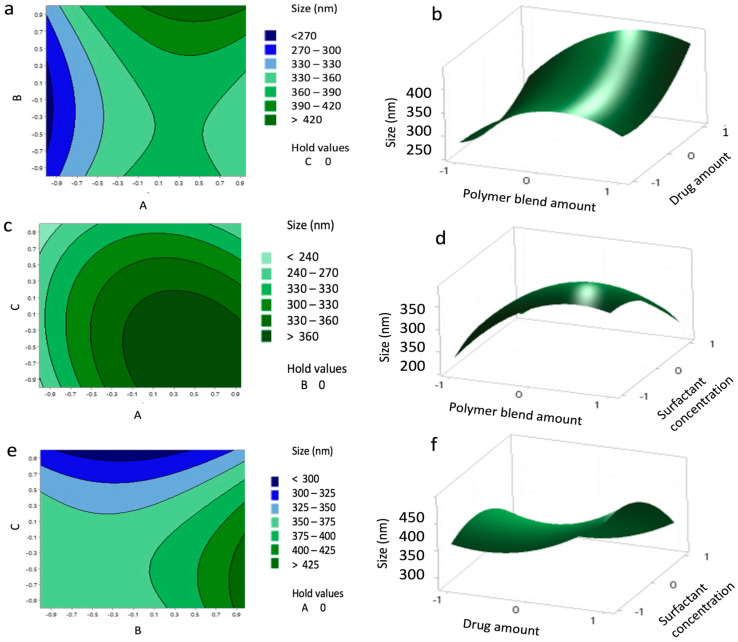
Contour plots (**a**,**c**,**e**) and surface plots (**b**,**d**,**f**) showing the effect of variables (A–C) on the size of the nanoparticles (NPs).

Contour and 3D surface plots (Figure 2) further illustrated these relationships and showed that interactions between independent variables were non-significant. The agreement between predicted and experimental values is presented in Table 7, confirming the reliability of the model.

### 3.3. BBD Predicted Model and Best Fit Regression Coefficient for Zeta Potential

Zeta potential responses were fitted via multiple regression to yield a significant polynomial model (Equation (3)), with R^2^ = 0.90, adjusted R^2^ = 0.85, and a non-significant lack of fit (*p* = 0.35). Regression analysis and ANOVA for NP zeta potential are summarized in Table 8 and Table 9, respectively

Zeta Potential (mV) = −26.205 − 4.196 A − 2.429 B + 2.550 C − 3.133 A*A + 1.717 B*C(3)

The model indicates that polymer and drug amounts exerted a significant antagonistic effect on surface charge, while surfactant concentration had a significant positive effect. The highest significant effect was shown to be the polymer amount, with 48.81% contribution.

**Figure 3:**
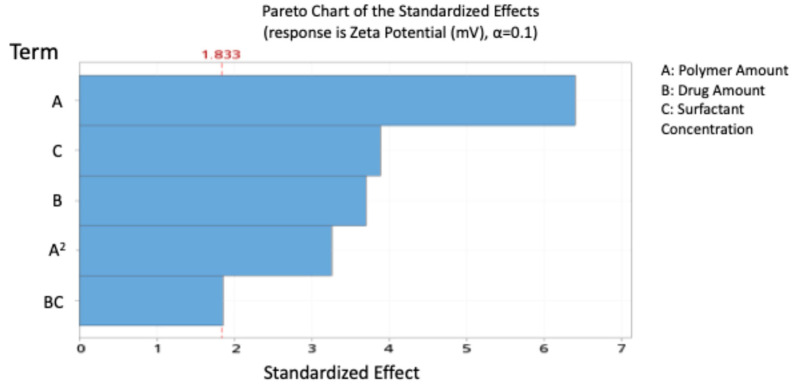
Pareto chart expressing the standardised effect of independent variables and their interaction on the charge of the nanoparticles (NPs).

The standardised effect ranking (Pareto chart, Figure 3) highlighted A, C, B, and A^2^ as the most influential factors on zeta potential.

**Figure 4:**
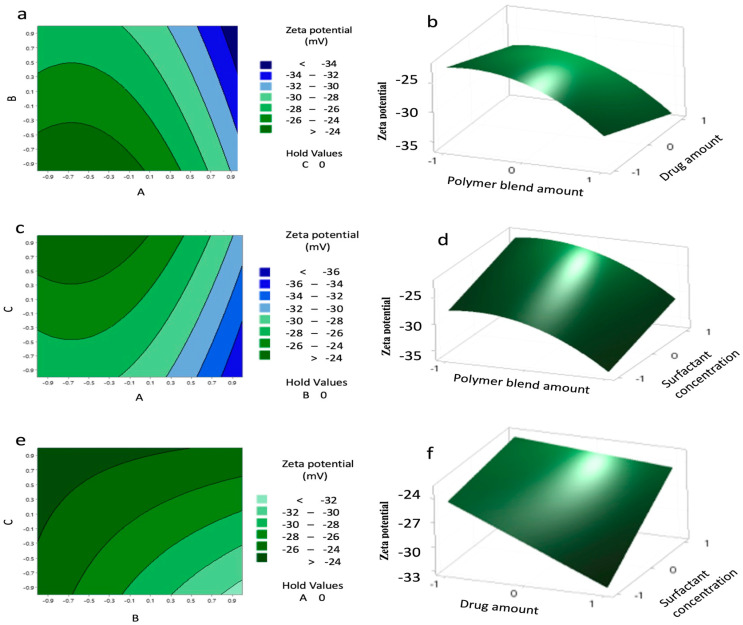
Contour plots (**a**,**c**,**e**) and surface plots (**b**,**d**,**f**) showing the effect of variables (A–C) on the charge of the nanoparticles (NPs).

Contour and 3D surface plots (Figure 4) further confirmed these trends, showing no significant A–B or A–C interactions, but a marked B–C interaction, where the highest negative charge occurred at B levels of 0.8–1 and C levels of –0.8 to –1. The agreement between predicted and experimental values (Table 10) confirms the reliability of the model.

### 3.4. BBD Predicted Model and Best Fit Regression Coefficient for EE%

The EE% responses were fitted via multiple regression to yield a significant second-order polynomial model (Equation (4)), with R^2^ = 0.92, adjusted R^2^ = 0.85, and a non-significant lack of fit (*p* = 0.62). Regression analysis and ANOVA for NP EE are summarized in Table 11 and Table 12, respectively

EE% = 56.72 + 2.771 A + 3.180 B + 3.029 C + 3.53 A*A + 3.69 B*B − 3.40 A*C + 3.19 B*C(4)

The model indicates that polymer amount, drug amount, and surfactant concentration positively influenced EE, while the polymer–surfactant interaction had a significant antagonistic effect. The drug amount had the highest significant effect on EE, with a percent of contribution of 20.50%.

**Figure 5:**
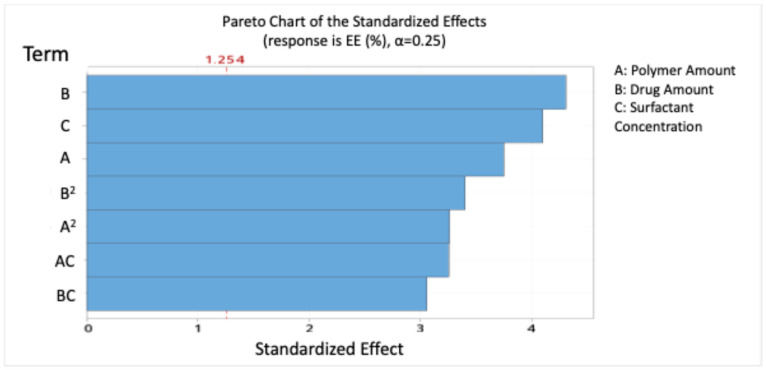
Pareto chart expressing the standardised effect of independent variables and their interaction on the encapsulation efficiency (EE%) of the nanoparticles (NPs).

Pareto analysis (Figure 5) confirmed that drug (B), surfactant (C), and polymer (A) were the most significant contributors to EE.

**Figure 6:**
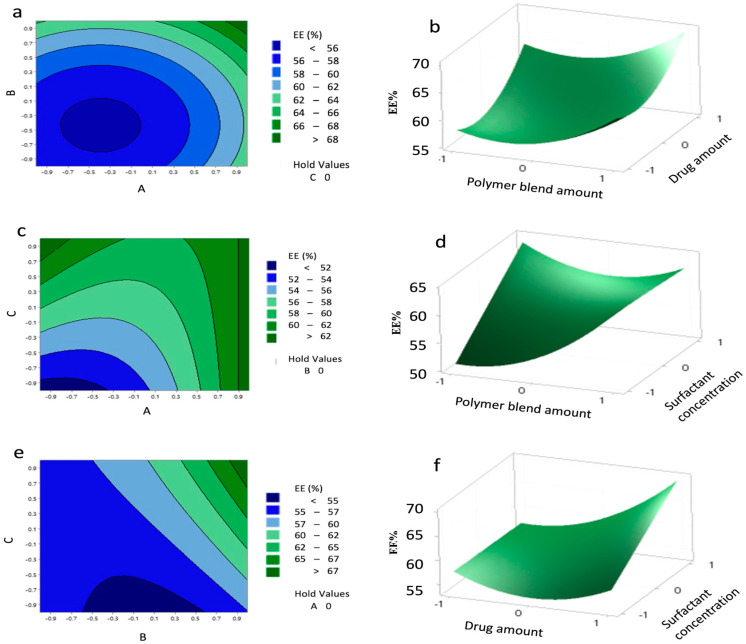
Contour plots (**a**,**c**,**e**) and surface plots (**b**,**d**,**f**) showing the effect of variables (A–C) on the encapsulation efficiency (EE%) of the nanoparticles (NPs).

Contour and 3D surface plots (Figure 6) revealed that EE above 62% could be achieved with polymer levels from 0.9 to 1 and surfactant levels from −1 to 1, and highlighted a significant interaction between drug and surfactant (B–C), while A–B interaction was non-significant. The agreement between predicted and experimental values (Table 13) confirms the reliability of the model.

## 4. Discussion

In the synthesis of polymeric NPs, it is important to carefully control their physicochemical properties, as these strongly affect both their EE for therapeutics and their behaviour in the body. For instance, surface characteristics influence interactions with proteins and cells, shaping biodistribution and cellular uptake, while particle size governs circulation time, tissue penetration, and clearance [15].

Polymer blends are gaining interest for drug delivery applications, where the compatibility of the polymers and their interaction with drugs can be used to control drug loading, which could be tailored depending on the ratios of polymers in the blend [16,17].

PCL can form miscible blends with other polymers, resulting in improved chemical characteristics as well as the solubility, degradation, and crystallinity of the polymer. This could promote the use of PCL as a tailored drug delivery vehicle with improved EE and release profiles [18,19]. The ratio of PCL with other polymers can be used to control their compatibility and drug permeability [20]. The degradation rate of PCL was enhanced and its hydrophobicity reduced when previously blended with PLGA and PLA [21].

Employing the double emulsion technique to develop polymeric NPs has great advantages for controlling the characteristics of the NPs [15].

Our results indicated that using a 50:50 ratio of PCL and PLGA blend produced monodisperse NPs with a higher EE (60.96% ± 6.62), compared to the 25:75 and 75:25 blends, with an acceptable particle size of 350 ± 3.52 nm, PDI of 0.36 ± 0.01, and charge of −32.50 ± 0.10 mV. The balanced ratio of PCL (hydrophobic, semi-crystalline) and the co-polymer (less hydrophobic, amorphous) likely promoted optimal miscibility and intermolecular interactions with the drug, reducing drug expulsion during emulsification and solidification. This synergistic effect minimised phase separation and enhanced drug–polymer compatibility, which explains the higher EE observed. These results are in accordance with other research that reported the highest EE with equal ratios of PCL/PLGA. PCL increased the hydrophobicity of the blends, helping to achieve higher EE of drugs. However, large particle sizes were reported when increasing the amount of PCL in the blend, which is likely due to certain chain organisation and entanglement [22]. This corroborates our result of a particle size of 451.10 ± 8.35 nm with the 75:25 PCL:PLGA ratio (Table 3).

As previously reported in our publication that despite the PCL advantages as a promising polymer for drug delivery, it still lacks effective approaches to enhance its encapsulation of drugs [23]. The goal of this study was to reduce the size and charge while maximising the EE% of the NP blend.

The size of the NPs was mainly influenced by the amount of polymers possibly due to increased viscosity and chain entanglement induced by higher polymer concentrations. However, the squared amount of polymers resulted in reduced particle size, while an increased surfactant concentration significantly reduced the size of the NPs due to a reduction in viscosity. Similar observations were reported by Petrova et al. (2024), who highlighted the influence of optimal polymer concentration on NP size uniformity [24].

The PCL/PLGA blend NPs exhibited a more negative zeta potential, indicating improved colloidal stability compared to PCL NPs, as described in our previous paper [25]. This can be attributed to the presence of more uncapped carboxylic groups in PLGA, which contribute to a higher magnitude of the negative surface charge [26]. A high magnitude of zeta potential, whether positive or negative, is crucial for maintaining distance between the NPs, preventing aggregation, and preserving stability [27]. Our model revealed that the charge of the NPs was significantly influenced by the amount of polymers and their amount squared, showing an antagonistic effect due to the higher content of carboxylic acid groups in the PCL/PLGA blend. Another study presented the influential role of the polymer functional groups on the overall charge of the NPs. That study suggested that acetate groups in polymeric chains contributed to the negatively charged NPs (−19.9 ± 0.4 mV) [28]. This elucidates that polymer concentration impacts the net charge on the surface of NPs.

The surfactant concentration had a significantly synergistic effect by reducing the magnitude of the negative charge, likely due to the shielding of the negatively charged polymers by the surfactant. However, at high PVA concentrations, interactions among PVA molecules can occur, leading to reduced NP stability [29]. Moreover, residual PVA adsorbed on the NP surface can contribute to an increase in particle size [30].

An ion pair HPLC method was employed to detect the efficiency of encapsulating IRH inside the matrix of the blend NPs. Ion-pair chromatography is particularly useful for hydrophilic and ionisable drug molecules because it improves retention and peak shape on reverse-phase columns by associating the drug with a counter-ion, thereby reducing early elution and interference from polar excipients. For example, Tang et al. (2022) demonstrated enhanced retention of polar anionic species via ion-pair chromatography in metabolomics workflows [31]. This supports our choice of the ion-pair method for IRH, which is hydrophilic and prone to poor retention under standard reversed-phase conditions. Our model implies that the EE% of the NPs is significantly influenced by the amount of drug. A significant increase in EE was also noticed upon increasing the amount of blended polymers which is likely due to an increase in chain entanglement, offering a larger matrix for EE. A high concentration of the surfactant also enhanced the EE. The interaction between the drug and surfactant helped maintain the drug entrapped in the NPs with higher levels. Whereas, the interaction between the polymer and surfactant negatively impacted the EE. As previously reported, a high concentration of surfactant could lead to the formation of networks between molecules of the surfactant and the chains of the polymers, leading to increased viscosity [32]. The amount of polymer and drug squared also enhanced the EE. Similarly to our results, a study reported that EE increased when the polymer amount (in their coded factor X_1_) increased [28].

In summary, high levels of the polymeric blend and drug resulted in the highest EE, confirming that the amount of blended polymers was sufficient to entrap a large amount of the drug.

Theoretical optimum conditions were determined by setting the desired characteristics of minimum particle size and charge, along with maximum EE. The optimum formulation factors were predicted to be achieved at level 1 of A, level 0.79 of B, and level 1 of C, corresponding to 162 mg, 8.37 mg, and 8%, respectively. Under these conditions, the model predicted NPs with a size of 283.06 nm, a zeta potential of −31.54 mV, and an EE of 70%.

The solvent evaporation emulsification method employed in this study is a well-established approach for NP preparation, with strong potential for scale-up. Scalability can be further enhanced through the integration of technologies such as microfluidics, which allow for precise control over particle size distribution, entrapment efficiency, and reproducibility at larger batch volumes. Importantly, the use of a BBD in this work provided a systematic framework to identify critical parameters and their interactions, enabling robust optimisation of particle size, charge, and entrapment efficiency. Such statistical modelling not only improves the reliability of the formulation at the laboratory scale but also supports scalability by ensuring that process adjustments can be predicted and controlled when moving to larger production volumes. While emerging techniques like microfluidics offer valuable opportunities for precise particle engineering and may complement future development, the robustness and statistical optimisation of the solvent evaporation emulsification process ensure that it remains a practical and scalable choice for translational applications.

Future work should focus on extending the physicochemical characterisation of the optimised polymeric blend NPs, including stability studies under different conditions and drug release kinetics. In addition, in vitro studies such as cytotoxicity assays and cellular uptake evaluations will provide insight into the safety profile and intracellular behaviour of the formulation. Ultimately, in vivo investigations assessing pharmacokinetics, biodistribution, and therapeutic efficacy will be crucial to confirm the translational relevance of the optimised NPs. Together, these follow-up studies will help bridge the gap between formulation optimisation and potential clinical application.

## 5. Conclusions

This study demonstrates that blending PCL with PLGA can be strategically employed to tailor NP properties, thereby enhancing their suitability as carriers for hydrophilic drugs such as IRH. Careful control of polymer ratios, drug amount, and surfactant levels was shown to significantly influence particle size, surface charge, and EE, with the balanced 50:50 PCL:PLGA blend achieving optimal physicochemical performance. Using the BBD, this study identified the predicted optimal formulation consisting of 162 mg polymer blend, 8.37 mg drug, and 8% surfactant, which is expected to yield NPs with a size of 283.06 nm, zeta potential of −31.54 mV, and EE of 70%. The application of BBD allowed systematic evaluation of the factors and their interactions, providing robust predictive models supported by regression and ANOVA analyses. These findings highlight the potential of polymer blends to improve EE while maintaining stability and desirable surface characteristics.

While the present work establishes a clear framework for optimising polymeric NP systems, further studies are required to advance translational relevance. In particular, extended physicochemical characterisation (including stability and drug release kinetics) as well as in vitro and in vivo validation are essential next steps to confirm safety, cellular uptake, and therapeutic efficacy. Overall, this study contributes valuable insights into the design of blended polymeric NPs and lays a foundation for their further development as versatile drug delivery systems.

## Figures and Tables

**Table 1:** Box–Behnken coded and actual values.

Variable	Coded Values		
	−1 (Low)	0 (Medium)	1 (High)
X1 = PCL-PLGA Amount (mg)	54 (27:27)	108 (54:54)	162 (81:81)
X2 = IRH Amount (mg)	3	6	9
X3 = PVA Concentration (%)	2	4	6
Y1 = size			
Y2 = Zeta Potential			
Y3 = EE			

**Table 2:** Box–Behnken 3-level factorial design with actual values.

Experiment	PCL-PLGA Amount (A; mg)	IRH Amount (B; mg)	PVA Concentration (C; %)
1	54	3	4
2	162	3	4
3	54	9	4
4	162	9	4
5	54	6	2
6	162	6	2
7	54	6	6
8	162	6	6
9	108	3	2
10	108	9	2
11	108	3	6
12	108	9	6
13	108	6	4
14	108	6	4
15	108	6	4

**Table 3:** Average particle size, polydispersity index (PDI), zeta potential, and encapsulation efficiency (EE%) of irinotecan hydrochloride (IRH) loaded blend nanoparticles (NPs) at various ratios of polymer amounts.

Formulation (PCL: PLGA)	Size (nm)	PDI	Zeta Potential (mV)	EE%
F1 (25:75)	234.26 ± 1.45	0.43 ± 0.01	−32.70 ± 0.34	59.69 ± 5.01
F2 (50:50)	350.23 ± 3.52	0.36 ± 0.01	−32.50 ± 0.10	60.96 ± 6.62
F3 (75:25)	451.10 ± 8.35	0.42 ± 0.03	−27.13 ± 0.35	56.85 ± 8.30

**Table 4:** Summary of particle size, polydispersity index (PDI), zeta potential, and encapsulation efficiency (EE%) of the prepared nanoparticle (NP) formulations used for Box–Behnken design (BBD) analysis.

Formulation No.	Size (nm) (*n* = 3)	PDI (*n* = 3)	Zeta Potential (mV) (*n* = 3)	EE% (*n* = 3)
1	313.80 ± 9.10	0.44 ± 0.02	−22.53 ± 2.23	58.48 ± 0.19
2	350.23 ± 3.52	0.36 ± 0.01	−32.50 ± 0.10	60.96 ± 6.62
3	296.37 ± 1.61	0.34 ± 0.03	−25.50 ± 0.50	66.25 ± 0.30
4	389.90 ± 2.52	0.93 ± 0.03	−36.70 ± 0.79	70.01 ± 0.22
5	221.60 ± 1.73	0.21 ± 0.21	−26.77 ± 2.63	49.76 ± 0.18
6	383.73 ± 5.65	0.48 ± 0.06	−35.30 ± 0.45	64.52 ± 0.06
7	206.03 ± 2.95	0.32 ± 0.04	−25.77 ± 0.64	62.82 ± 0.08
8	250.26 ± 2.31	0.54 ± 0.05	−29.63 ± 3.62	63.99 ± 0.26
9	338.63 ± 6.08	0.35 ± 0.05	−25.37 ± 0.47	58.54 ± 0.21
10	461.33 ± 6.49	0.34 ± 0.05	−34.93 ± 0.70	56.46 ± 0.06
11	284.27 ± 7.51	0.32 ± 0.08	−21.93 ± 0.55	58.00 ± 0.49
12	354.93 ± 3.32	0.42 ± 0.05	−24.63 ± 0.35	68.70 ± 0.05
13	380.87 ± 9.5	0.44 ± 0.04	−27.07 ± 0.28	55.56 ± 0.11
14	341.37 ± 1.39	0.60 ± 0.01	−24.97 ± 0.92	59.27 ± 0.09
15	367.33 ± 3.34	0.45 ± 0.04	−24.53 ± 0.70	55.26 ± 0.09

**Table 5:** Summary of regression coefficient analysis for the response Y1 (size).

Coefficient	β0	β1	β2	β3	β4	β5	β6	β7	β8	β9
Size	363.18	42	26.90	−38.70	−60	34.40	−37.80	14.30	−29.50	−13
*p* value	0.000	0.014	0.065	0.019	0.016	0.096	0.074	0.417	0.127	0.457

**Table 6:** Degree of freedom (DF), sum of squares (SS), mean square (MS), F-statistic (*F*-Value), and Probability value (*p* value) for the size of the nanoparticles (NPs).

Regression	DF	SS	MS	*F* Value	*p* Value
Size (nm)	9	60,682.40	6742.50	6.47	0.02

**Table 7:** Experimental and theoretical values with residuals of the response Y1.

Formulation No.	Experimental (Observed) Value of Size	Theoretical (Predicted) Value of Size	Residuals	%Error
1	313.80	282.86	30.94	9.86
2	350.23	338.39	11.84	3.38
3	296.37	308.21	−11.84	−4.00
4	389.90	420.84	−30.94	−7.94
5	221.60	232.61	−11.01	−4.97
6	383.73	375.65	8.08	2.11
7	206.03	214.12	−8.08	−3.92
8	250.26	239.24	11.02	4.40
9	338.63	358.56	−19.93	−5.88
10	461.33	438.48	22.86	4.95
11	284.27	307.12	−22.86	−8.04
12	354.93	335.01	19.93	5.61
13	380.87	363.19	17.68	4.64
14	341.37	363.19	−21.82	−6.39
15	367.33	363.19	4.14	1.13

**Table 8:** Summary of regression coefficient analysis for the response Y2 (Zeta potential).

Coefficient	β0	β1	β2	β3	β4	β5
Zeta Potential (mV)	−26.20	−4.19	−2.42	2.55	−3.13	1.71
*p* value	0.000	0.000	0.005	0.004	0.010	0.098

**Table 9:** Degree of freedom (DF), sum of squares (SS), mean square (MS), F-statistic (*F*-Value), and Probability value (*p* value) for the charge of the nanoparticles (NPs).

Regression	DF	SS	MS	*F* Value	*p* Value
Charge	5	288.49	57.69	16.72	0.000

**Table 10:** Experimental and theoretical values with residuals of the response Y2.

Formulation No.	Experimental (Observed) Value of Charge	Theoretical (Predicted) Value of Charge	Residuals	%Error
1	−22.53	−22.71	0.18	−0.80
2	−32.50	−31.10	−1.40	4.29
3	−25.50	−27.57	2.07	−8.12
4	−36.70	−35.96	−0.74	2.01
5	−26.77	−27.69	0.92	−3.46
6	−35.30	−36.08	0.78	−2.22
7	−25.77	−22.59	−3.18	12.32
8	−29.63	−30.98	1.35	−4.56
9	−25.37	−24.61	−0.76	2.99
10	−34.93	−32.90	−2.03	5.82
11	−21.93	−22.94	1.01	−4.60
12	−24.63	−24.37	−0.27	1.08
13	−27.07	−26.20	−0.86	3.18
14	−24.97	−26.20	1.24	−4.96
15	−24.53	−26.20	1.67	−6.81

**Table 11:** Summary of regression coefficient analysis for the response Y3 (encapsulation efficiency (EE%)).

Coefficient	β0	β1	β2	β3	β4	β5	β8	β9
EE%	56.72	2.77	3.18	3.02	3.53	3.69	−3.40	3.19
*p* value	0.000	0.007	0.004	0.005	0.014	0.011	0.014	0.018

**Table 12:** Degree of freedom (DF), sum of squares (SS), mean square (MS), F-statistic (*F*-Value), and Probability value (*p* value) for the encapsulation efficiency (EE%) of the nanoparticles (NPs).

Regression	Df	SS	MS	*F* Value	*p* Value
EE%	7	393.19	56.17	12.84	0.002

**Table 13:** Experimental and theoretical values with residuals of the response Y3.

Formulation No.	Experimental (Observed) Value of EE%	Theoretical (Predicted) Value of EE%	Residuals	%Error
1	58.48	57.99	0.49	0.84
2	60.96	63.53	−2.57	−4.22
3	66.25	64.35	1.90	2.87
4	70.01	69.89	0.12	0.17
5	49.76	51.06	−1.30	−2.61
6	64.52	63.39	1.13	1.74
7	62.82	63.91	−1.09	−1.74
8	63.99	62.66	1.33	2.08
9	58.54	57.39	1.15	1.96
10	56.46	57.36	−0.90	−1.60
11	58.00	57.06	0.94	1.62
12	68.70	69.81	−1.11	−1.62
13	55.56	56.72	−1.16	−2.09
14	59.27	56.72	2.55	4.30
15	55.26	56.72	−1.46	−2.64

## Data Availability

The datasets and materials used and analysed during the current study are available from the corresponding author upon request.
